# Networking Feasibility of Quantum Key Distribution Constellation Networks

**DOI:** 10.3390/e24020298

**Published:** 2022-02-20

**Authors:** Junyong Wang, Liang Chang, Hongyu Chen, Zhencai Zhu

**Affiliations:** 1 Innovation Academy for Microsatellites, Chinese Academy of Sciences, Shanghai 201203, China; changl@microsate.com (L.C.); chenhy@microsate.com (H.C.); zczhu@hotmail.com (Z.Z.); 2 University of Chinese Academy of Sciences, Beijing 100049, China; 3 School of Information Science and Technology, ShanghaiTech University, Shanghai 201210, China

**Keywords:** decoy-state method, satellite constellation, low-earth orbit satellite, networking

## Abstract

Quantum key distribution constellation is the key to achieve global quantum networking. However, the networking feasibility of quantum constellation that combines satellite-to-ground accesses selection and inter-satellite routing is faced with a lack of research. In this paper, satellite-to-ground accesses selection is modeled as problems to find the longest paths in directed acyclic graphs. The inter-satellite routing is interpreted as problems to find a maximum flow in graph theory. As far as we know, the above problems are initially understood from the perspective of graph theory. Corresponding algorithms to solve the problems are provided. Although the classical discrete variable quantum key distribution protocol, i.e., BB84 protocol, is applied in simulation, the methods proposed in our paper can also be used to solve other secure key distributions. The simulation results of a low-Earth-orbit constellation scenario show that the Sun is the leading factor in restricting the networking. Due to the solar influence, inter-planar links block the network periodically and, thus, the inter-continental delivery of keys is restricted significantly.

## 1. Introduction

The communication security is extremely important in commerce, finance and government affairs. Quantum key distribution (QKD) [1] is a promising technology to protect communication security in times when the security of the encryption system based on mathematical complexity is imperiled under the quantum computing system. QKD uses the characteristics of quantum mechanics to ensure communication security. It enables both sides of communication to generate and share a random and secure key to encrypt and decrypt messages. With the principle of one time pad (OTP), QKD ensures absolute security, theoretically. In the developing stages of QKD, there were many experiments performed to inspect the technology of QKD. The DARPA quantum network [2,3] is a 10-node quantum key distribution network. It supports a standards-based internet computer network protected by quantum key distribution. Secure Communication based on Quantum Cryptography (SECOQC) [4,5] is a project that aims to develop quantum cryptography. The network uses standard optics fibers to interconnect six nodes and eight links of the multi-protocol type. However, the key distribution rate using optical fibers is restricted by channel losses that increase exponentially at about 0.2 dB/km in optical fiber. Owing to the high loss of optics fibers, the terrestrial relay system can hardly guarantee security without quantum repeaters, which are still in the experiment stage.

Therefore, to achieve the goal of distributing quantum keys globally, it has to establish the constellation of a quantum satellite [6,7] instead of the terrestrial relay system. For the space QKD, variable loss channels [8,9] are the important factors to be taken into consideration. Additionally, the geometrical loss of beams is too high to decrease the distances of satellite-to-ground QKD. The constellation consisting of low-Earth-orbit (LEO) satellites is the practical choice to build inter-continental quantum networks. Before building such networks, the networking feasibility of QKD constellation networks is the primary crux of the matter. In this paper, regarding the networking feasibility issue, the following two aspects are researched.

- Accesses selection of satellite-to-ground QKD.
- Inter-satellite routing of forwarding quantum keys.

For satellites (ground stations), there may be multiple ground stations (satellites) in their sights. Satellite-to-ground accesses are restricted due to constraints, e.g., number of payloads, and status of payloads. Access selection is to plan the satellite-to-ground accesses during operations. By selecting the proper accesses for the quantum constellation, more keys can be distributed to each ground station. For pursuing better performance of the quantum constellation, accesses selection is the key problem to be solved. There are a few studies focused on this aspect [10,11,12]. Vergoossen et al. [10] modeled the QKD constellation. The pointing conditions of the QKD optical device are not taken into consideration in their research and the accesses selection method is simple. Polink et al. [11] researched the scheduling of satellites in a tiny QKD constellation that only works for a small region. Wang et al. [12] investigated the performance of a QKD constellation that consists of satellites in a single orbital plane, including the accesses selection algorithm, key re-distribution algorithm and relayed-key consumption.

Quantum keys are used to transfer messages securely on the internet only when the keys are distributed to a couple of ground stations. To distribute quantum keys in a large-scale region, they are transferred via inter-satellite links from one satellite to another. However, the transmissions are encrypted by using quantum keys generated by inter-satellite links. Due to the principle of OTP, the quantum keys are discarded after being used. Therefore, the capabilities of inter-satellite links are restricted. Inter-satellite routing calculates the forwarding path of quantum keys according to the given sending and receiving satellites and the capabilities of inter-satellite links. Proper inter-satellite routing helps the quantum constellation to distribute more quantum keys with less consumption of quantum keys shared between inter-satellites. Currently, most studies focus on the routing on terrestrial QKD networks [13,14,15]. There are a few articles about the routing of the QKD constellation [16]. Wang et al. [16] researched the routing algorithm and key resource allocation of QKD satellite networks. Both of their models to calculate the key rate and inter-satellite links are rough, as they did not take the finite-size effect and secure key distillation into consideration.

The paper is organized as follows. Section 1 is the introduction. Section 2 elaborates the fundamentals of QKD and the principles of the QKD constellation. Section 3 illustrates access selection within graph models. Section 4 expounds the inter-satellite routing algorithm with graph models and provides the traffic balance algorithm. Section 5 shows the simulation results. Section 6 is the discussion.

## 2. Fundamentals of QKD and Principles of QKD Constellation

In this section, the fundamentals of QKD and the principles of the QKD constellation are elaborated.

### 2.1. Fundamentals of QKD

There are several QKD schemes, e.g., discrete variable QKD (DV-QKD) and continuous variable QKD (CV-QKD) [17,18]. The fundamentals of different QKD schemes are similar. QKD uses two channels, including quantum link and classical link, to generate quantum keys between two parties. Quantum links are used to transmit and receive random quantum signals, and classical links share the information that is utilized in the post-processing procedure. Firstly, the communication parties generate a large number of quantum key bits as raw bits through quantum links. Secondly, these raw bits are sifted according to the information shared between them through classical links. Lastly, they further distill the secure key bits from the sifted bits by the technology of privacy amplification. Therefore, by the use of an OTP strategy with two parties, unconditional security is guaranteed.

### 2.2. Principles of QKD Constellation

**Figure 1:**
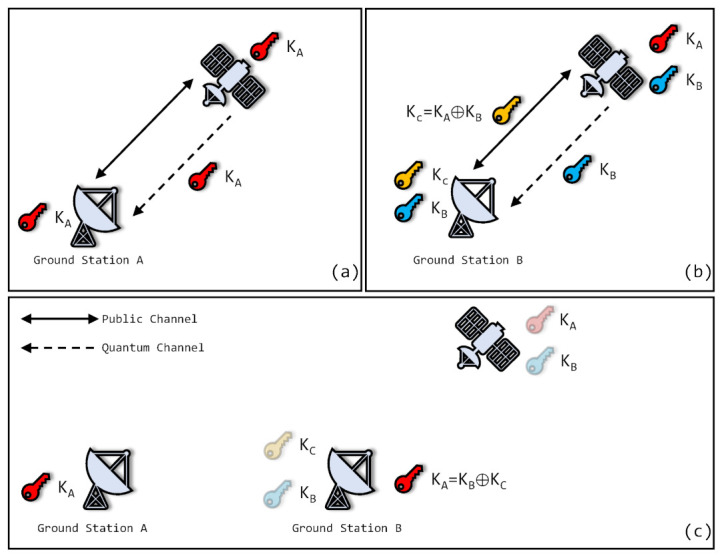
The storing and forwarding of quantum keys. (**a**): Firstly, satellite visits ground station A. Key $K_{A}$ is generated between them by quantum link; (**b**): Secondly, satellite visits ground station B. Key $K_{B}$ is generated between them by quantum link. Then, key $K_{C}$ is generated by XOR operation of $K_{A}$ and $K_{B}$ by satellite and is sent by classical link. Ground station B decrypts $K_{C}$ to obtain key $K_{A}$; (**c**): Finally, ground stations A and B share same key $K_{A}$.

Figure 1 shows the storing and forwarding of quantum keys without inter-satellite relay. Quantum keys $K_{A}$ and $K_{B}$ are generated when a satellite passes ground stations *A* and *B*, respectively. $K_{A}$ are stored in the satellite and are distributed to *B*. To distribute $K_{A}$ securely, encrypted key $K_{C}$ is delivered to *B*. $K_{C}$ is the key encrypted by $K_{A}$ and $K_{B}$. A simple encryption scheme is XOR operation: $K_{C}\;=\;K_{A}⊕K_{B}$. When it receives $K_{C}$, ground station *B* decrypts $K_{A}\;=\;K_{C}⊕K_{B}$. Here, a distribution of quantum keys is completed.

The delay of quantum keys without inter-satellite relay depends on the accessing interval between two ground stations. For LEO satellites, the delay is several days.

**Figure 2:**
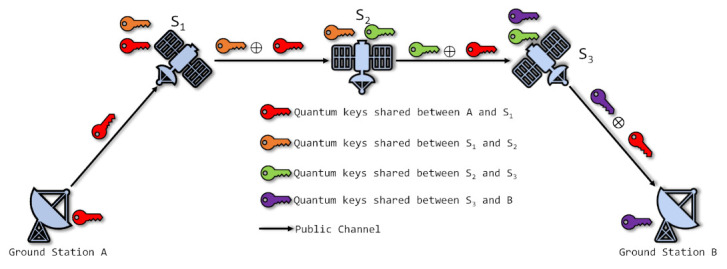
The inter-satellite relay of quantum keys. All the quantum keys are generated by quantum channels (not drawn in figure) in advance. The red quantum keys are delivered from one ground station to another. Other quantum keys are discarded after red keys have been relayed.

By inter-satellite relaying, the quantum keys are routed to the satellite near the target ground station. In addition, by routing the quantum keys from other satellites, the size of key $K_{C}$ is increased. Therefore, with the help of inter-satellite relay, not only does the delay of quantum keys decrease, but the keys distributed to the ground stations increase. Figure 2 describes the inter-satellite relay of quantum keys. The quantum key to be delivered is encrypted and decrypted hop by hop so that the security is assured. All the keys used to be encrypted and decrypted are generated by QKD.

The inter-satellite QKD is divided into inter-planar QKD and intra-planar QKD. Inter-planar QKD is performed by the satellites distributed in two different and adjacent orbital planes. Meanwhile, intra-planar QKD is processed by adjacent satellites distributed in the same orbital planes.

As the result of the principle of OTP, quantum keys of inter-satellite links are discarded after the transfers are complete. Hence, the quantum keys of inter-satellite links are bottlenecks that limit inter-satellite relaying. The recoveries of quantum keys depend on the generated key rate of inter-satellite QKD.

## 3. Accesses Selection

Due to imbalanced distribution of ground stations, there are numerous ground stations located in an area, for example, Europe and Southeast Asia. When passing such an area, a satellite has to discard opportunities to generate quantum keys with some ground stations because of insufficient QKD payloads. As the motion constraints of telescope pointing state, opportunities with some ground stations are mutually exclusive, i.e., a telescope cannot point to its target one by one. Accesses selection is the decision of whether or not a satellite accesses a ground station.

### 3.1. Assumptions

To simplify the problem, accesses selection is based on the following assumptions:

- Meteorological conditions of each ground station remain constant during one access.
- Accesses between satellites and ground stations are available only when both the satellites and ground stations are in nights.

As link losses are significantly affected by meteorological conditions [19,20,21], it is essential to take meteorological conditions into consideration. The first assumption denotes that meteorological conditions change per access. As the meteorological system is chaotic, the meteorological conditions are predictable only in the short term. The smaller the period is, the more accurate the prediction would be. To simplify the effects of the meteorological system, meteorological conditions are deemed constant during one access.

As link losses under sunlight are about 10 dB greater than those under nights [22], it is unavailing to utilize quantum links under sunlight. Therefore, in this paper, accesses are available when both satellites and ground stations are in nights.

### 3.2. Pointing Constraint

The main constraint of accesses selection is the pointing constraint. The transmission process for QKD in free-space is different than a standard transmission. For both satellite-to-ground and inter-satellite QKD, the optical channels must be established before the transmission process for QKD. Such a process may cost several seconds. Hence, the telescopes of the transmitter and receiver should aim each other as soon as possible. Otherwise, there are barely any photons to be emitted to the receiver during the turning of the telescopes. In the QKD procedures, both the telescopes aim each other in the whole QKD procedure so that the beams collimate. To simplify the problem, it can be deemed that the telescopes always aim each other in the procedures. When the procedures are complete, the telescopes need point to their next targets before the next QKD procedures start. Once the telescopes cannot point to targets in time, the QKD procedures are delayed.

### 3.3. Formulations

Table 1 lists the definitions of symbols for interpreting the problem. The starting time $st_{i}$ and ending time $et_{i}$ are the minimum and maximum times when satellite $s_{i}$ and ground station $g_{i}$ are in each other’s sights in the access $a_{i}$. $k_{i}$ is the key size generated of the access $a_{i}$ in a clear night. The function $T(p_{1},p_{2})$ denotes the minimum transition time from the pointing state $p_{1}$ to $p_{2}$.

In the Table 1, the calculation of $T(p_{1},p_{2})$ references the bang–bang control [23]. The following conditions are all satisfied if $F(a_{i},a_{j})\;=\;1$:

- $Index\left(a_{i}\right)\;\neq \;Index\left(a_{j}\right)$.
- $Sat\left(a_{i}\right)\;=\;Sat\left(a_{j}\right)$ or $Gnd\left(a_{i}\right)\;=\;Gnd\left(a_{j}\right)$.
- $St\left(a_{j}\right)\;>\;Et\left(a_{i}\right)$.
- If $Sat\left(a_{i}\right)\;=\;Sat\left(a_{j}\right)$ such that $T\left(Fps\left(a_{i}\right),Ips\left(a_{j}\right)\right)\leq St\left(a_{j}\right)\;-\;Et\left(a_{i}\right)$.
- If $Gnd\left(a_{i}\right)\;=\;Gnd\left(a_{j}\right)$ such that $T\left(Fpg\left(a_{i}\right),Ipg\left(a_{j}\right)\right)\;\leq \;St\left(a_{j}\right)\;-\;Et\left(a_{i}\right)$.

Otherwise, $F(a_{i},a_{j})\;=\;0$.

The first condition denotes that accesses $a_{i}$ and $a_{j}$ are not the same access; The second condition indicates accesses $a_{i}$ and $a_{j}$; The third condition represents that the starting time of access $a_{j}$ is greater than the ending time of $a_{i}$; The fourth and fifth conditions mean that, for the common satellite or ground station of $a_{i}$ and $a_{j}$, there is enough time to transit from the former pointing state to the latter.

### 3.4. Graph Model

**Figure 3:**
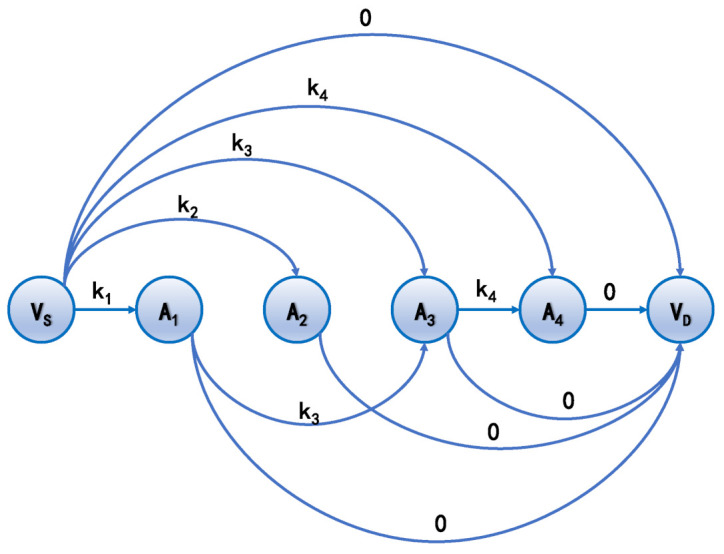
The accesses selection graph. The capacities of edges are the keys of the corresponding accesses represented by the ending vertices. For ending vertex $V_{D}$, the related capacities are all 0. Note that the ground station cannot implement both accesses $A_{1}$ and $A_{2}$. The similar conditions include $\left(A_{2},A_{3}\right)$, $\left(A_{1},A_{4}\right)$ and $\left(A_{2},A_{4}\right)$. The source vertex $V_{S}$ can reach any vertices, except itself, within one hop. The ending vertex $V_{D}$ can be reached by any vertices, except itself, within one hop. The solution of accesses selection is to find a path that starts from $V_{S}$ to $V_{D}$ with a maximum sum of capacities of edges.

Here, the graph model of the accesses selection and how to select the accesses in the way of applying graph theory are introduced. Graph model G = (V,E) is a directed acyclic graph (DAG), where *V* is a set of vertices and *E* a set of edges. Hereby, *V* denotes the indices of accesses, and *E* denotes whether the transition from one access to another is feasible. To make the accesses selection simpler, only the accesses that are implemented by same ground station are considered in one graph. To make the prediction of meteorological condition more accurate, the starting time and ending time of the accesses are bounded in given time interval $\left[t_{0},t_{1}\right]$. Assume that graph *G* is about the ground station gnd. The vertices set $V_{0}\;=\;\left\{Index\left(a\right)|Gnd\left(a\right)\;=\;gnd,t_{0}\;\leq \;St\left(a\right)\;\leq \;t_{1}\right\}$ represents the accesses that are to be selected. Auxiliary vertices $V_{S}$ and $V_{D}$ are added, which are the source vertex and destination vertex, respectively. $V_{S}$ is deemed as the index of the last accesses of the ground station gnd whose starting time is less than $t_{0}$. $V_{D}$ is the virtual vertex that represents a terminate of the path. Therefore, $V\;=\;V_{0}\cup \left\{V_{S},V_{D}\right\}$. The condition that there is an edge $(Index\left(a_{i}\right),Index\left(a_{j}\right))\in E$ is $F(a_{i},a_{j})\;=\;1$. Furthermore, there are edges $\left(V_{S},Index\left(a\right)\right),a\;\in V_{0}$ and $\left(Index\left(a\right),V_{D}\right)$. For the incoming edges of Index(a), the capacities are set to be Key(a). For the incoming edges of $V_{D}$, the capacities are 0. Figure 3 is a graph example of the accesses selection.

The accesses selection is to find a path with a maximum sum of weighted values from the source node $V_{S}$ to destination node $V_{D}$. For example, in Figure 3, if the path with a maximum sum of weighted values is $\left(V_{S},A_{1},A_{3},A_{4},A_{D}\right)$, then the ground station can select accesses $A_{1},A_{3},A_{4}$ to implement for maximizing the distributed keys during the time interval $\left[t_{0},t_{1}\right]$. Such a problem is solved by dynamic programming. Hence, by maximizing the sum of weighted values of the path, the solution of the problem finds a selection of accesses with the same ground stations in a given time interval. However, the accesses selection of the constellation needs to be studied further.

### 3.5. Solving Algorithm

To solve the accesses selection of the constellation, the feasibilities between the accesses with same satellites are taken into consideration. The accesses selection algorithm of constellation is proposed in Algorithm 1. Note that $\left(i_{1},i_{2},\cdots ,i_{N_{G}}\right)$ in line 8 is the index sequence of the ground station in ascending order of the distributed key sizes. Hence, in lines 14 and 15 of Algorithm 1, the $i_{j}$-th ground station is processed in the *j*-th loop.
**Algorithm 1** Accesses selection algorithm of constellation.**Input:** Set of accesses *A*, set of ground stations *G*, planning interval *T*, planning time $T_{p}$.**Output:** Implemented set of accesses $A^{*}$
    1: **for**
i = 1 **to** $N_{G}$
**do**
    2:      Initialize the implemented set of accesses of ground station *i*, $A_{i}^{*}\leftarrow \emptyset$
    3:      Initialize the distributed key sizes of ground station *i*, $k_{i}\leftarrow 0$
    4: **end for**
    5: t←0
    6: **while**
$t\leq T_{p}$
**do**
    7:      Find the set of accesses $A^{\prime }$ that are in the time interval [t,t + T], $A^{\prime }\leftarrow \left\{a|t\;\leq \;St(a)\;\leq \;t\;+\;T,a\;\in A\right\}$.
    8:      Obtain the sequence of distributed key sizes by ascending order $\left(i_{1},i_{2},\cdots ,i_{N_{G}}\right)$, where $k_{i_{1}}\;\leq \;k_{i_{2}}\;\leq \;\cdots \;\leq \;k_{i_{N_{G}}}$.
    9:      **for** j = 1 **to** $N_{G}$ **do**
    10:        Find all the set of accesses $A_{i_{j}}^{\prime }$ about the ground station $i_{j}$ from $A^{\prime }$, $A_{i_{j}}^{\prime }\leftarrow \left\{a|Gnd\left(a\right)\;=\;i_{j},a\;\in A^{\prime }\right\}$.
    11:        Generate graph *G*.
    12:        Calculating the longest path by dynamic programming $P^{*}$.
    13:        Add the accesses in the longest path into implemented set of accesses, $A^{*}\leftarrow A^{*}\cup P^{*}$.
    14:        Update the distributed key sizes of ground station $i_{j}$, $k_{i_{j}}\leftarrow \sum_{a\in P^{*}}Key\left(a\right)$.
    15:        Delete the accesses in $A^{\prime }$ conflicting with $P^{*}$, $A^{\prime }\leftarrow A^{\prime }\setminus \left\{a|F\left(a,b\right)\;=\;0\;\mathrm{or}\;F\left(b,a\right)\;=\;0,\mathrm{if}\;Sat\left(a\right)\;=\;Sat\left(b\right),\forall b\in P^{*},\forall a\in A^{\prime }\right\}$.
   16:      **end for**
   17:      t←t + T
    18: **end while**


## 4. Inter-Satellite Routing

Routing of the QKD constellation comprises forwarding quantum keys by inter-satellite links that generate quantum keys continuously. Quantum keys of inter-satellite QKD are consumed to encrypt or decrypt quantum keys of satellite-to-ground QKD forwarded along links and then discarded due to the OTP principle. The critical problem is how to forward quantum keys. It is divided into two aspects: firstly, updating the network status among the QKD constellation, and secondly, calculating a forwarding path.

### 4.1. Formulations

Table 2 lists the definitions of symbols that are used in inter-satellite routing.

In Table 2, Hop is the minimum hop count from satellite $s_{i}$ to $s_{j}$; $Key^{ISL}\left(s_{i},s_{j}\right)$ is the quantum key sizes stored at satellites $s_{i}$ and $s_{j}$ if there is an inter-satellite link (ISL) established between them; $Key^{Pool}\left(s,g\right)$ is the quantum key sizes stored at satellite *s* and ground station *g*. Once they are delivered to another ground station, they are eliminated from the key pools of ground station *g* and satellite *s*.

### 4.2. Network Status Update

The first step is to design an information sharing mechanism so that the satellites understand the networks of QKD constellation before forwarding the quantum keys. The network status of the QKD constellation is the key pool status of each satellite and is maintained by each satellite. The key pool consists of the sizes of quantum keys of satellite-to-ground QKD and inter-satellite QKD. For each satellite, the number of inter-satellite links is 4, i.e., front and back sides (for intra-planar links) and left and right sides (for inter-planar links). The number of quantum keys of satellite-to-ground QKD is equal to the number of ground stations. Assume that there are $N_{gnd}$ ground stations, and $N_{sat}$ satellites. The size of each key pool status is $N_{gnd}\;+\;4$. For the whole constellation, the total size of the key pool status is $N_{sat}\;\times \;\left(N_{gnd}\;+\;4\right)$. Flooding the messages of the key pool status without restriction certainly blocks the networks. For the purpose of saving network traffic and avoiding network blocks, the information sharing mechanism proposed in this paper is based on the following principles.

1. Full update disseminates periodically.
2. Incremental update disseminates when the key pool status is changed.
3. The information dissemination direction of the satellites in the same plane is adjacent to the satellites of the next hop, and that in the adjacent plane is adjacent to the satellites of the other plane.

#### 4.2.1. Full Update

A full update is necessary to synchronize the key pool status of each satellite. As the dropped packet occurs randomly, for each satellite, differences of the key pool status of constellation appear after a long term. Under such conditions, after calculating the forwarding path, the forwarding of quantum keys may fail due to the lack of inter-satellite quantum keys. However, due to the heavy burden of network traffic, it is not advisable for satellites to broadcast the full status of the key pool within a short period.

#### 4.2.2. Incremental Update

Incremental updates are the key to synchronize the key pool status among constellations. Satellites broadcast their changes of the key pool status when new quantum keys are stored or the stored quantum keys are consumed. As the network traffic is small, incremental updates can hardly block the network.

#### 4.2.3. Dissemination Direction

**Figure 4:**
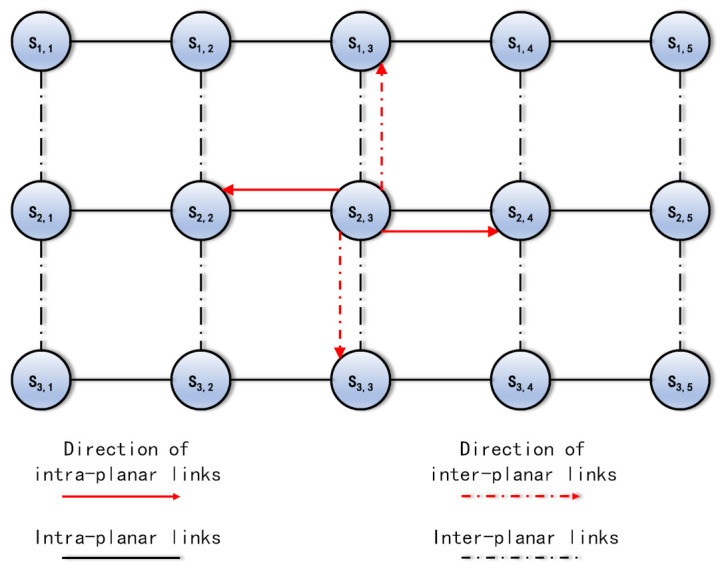
The network status dissemination direction.

If a satellite disseminates its network status, it broadcasts the messages to all the adjacent satellites. Other satellites receive the messages and update their network status table. For the satellites that are in the same plane, the messages are delivered to the other adjacent satellites along the intra-planar links. For the satellites that are in different planes, the messages are delivered to the other adjacent satellites along the inter-planar links. The information dissemination direction is depicted in Figure 4.

### 4.3. Forwarding Path

The second step is to calculate a path in which the quantum keys are forwarded properly. In this paper, the forwarding path is represented by a graph model and calculated by the Ford–Fulkerson method.

Graph G = (V,E) consists of a set of vertices *V* and edges *E*. The vertex $V_{i}$ is represented by satellite $s_{i}$. For the vertices $V_{i},V_{j}$, the condition that there is an edge $(V_{i},V_{j})\in E$ is that there is an inter-satellite link from satellite $s_{i}$ to $s_{j}$. The corresponding capacity is $C_{V_{i},V_{j}}\;=\;Key^{ISL}\left(s_{i},s_{j}\right)$, i.e., the quantum keys stored by the inter-satellite links.

Physically, quantum key flows start from satellites (may be multiple source nodes) to another satellite (sink node). The source nodes are the satellites that store the quantum keys, and the sink node is the satellite that consumes the quantum keys. Actually, as the required quantum keys may be stored at several satellites, the number of source nodes is probably several. Meanwhile, the number of sink nodes is only one, as there is only one satellite that accesses the ground station to distribute quantum keys. Therefore, quantum key flows consist of multiple single quantum key flows that end at the same node.

**Figure 5:**
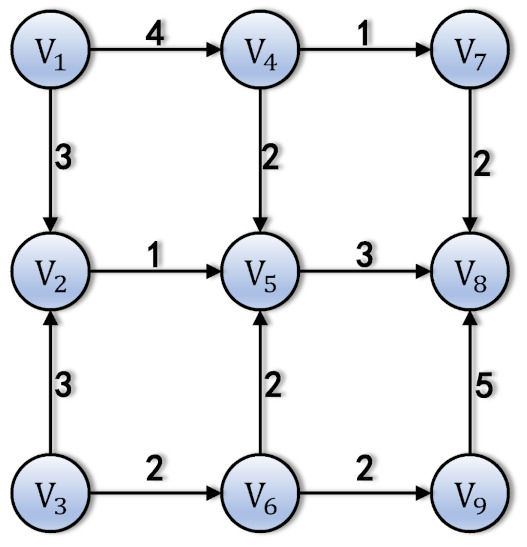
The network graph. $V_{8}$ is the sink node. The edges always start from the nodes with greater hops and ends at smaller ones. The numbers of edges are the capacities of links.

Assume that graph *G* is shown as Figure 5. The directions of the edges are fixed, as the sink satellite is $s_{8}$. The status of the constellation’s key pools are described in Table 3.

If satellite $s_{8}$ is accessing ground station $g_{3}$, the quantum keys of ground stations $g_{1}$ and $g_{2}$ need to be distributed to ground station $g_{3}$ by satellite $s_{8}$. Hence, satellite $s_{8}$ distributes the quantum keys of ground stations $g_{1}$, $g_{2}$ and $g_{3}$ with key sizes $K_{1}$, $K_{2}$ and $K_{3}$, correspondingly. Note that $K_{3}\;=\;K_{1}\;+\;K_{2}$ because of the requirements of encryption and decryption. Thus, the quantum keys are distributed safely, without waste. However, there may not be enough quantum keys stored by satellite $s_{8}$. Then, the other satellites forward their own quantum keys to $s_{8}$ by inter-satellite links to compensate. As the forwarded quantum keys consume the quantum keys generated by inter-satellite links, the capacities of each inter-satellite link restrict the quantum key flows. In addition, in such a graph, as there are several satellites that provide quantum keys, it is difficult to directly calculate the quantum key flows that maximize the sizes of the forwarded keys and satisfy the constraints of the inter-satellite links.

**Figure 6:**
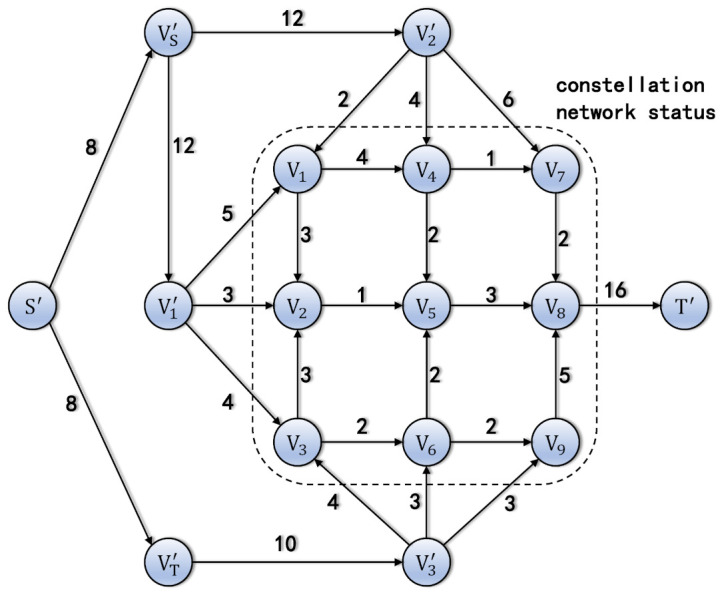
The auxiliary graph. The graph *G* consists of the vertices and edges in the dashed circle.

To solve the above problem, the auxiliary graph $G^{\prime }\;=\;\left(V^{\prime },E^{\prime }\right)$ illustrated in Figure 6 is proposed. The auxiliary graph adds additional vertices $V_{1}^{\prime },V_{2}^{\prime },V_{3}^{\prime },V_{S}^{\prime },V_{T}^{\prime },S^{\prime }$ and $T^{\prime }$. The capacities of the edges from $V_{i}^{\prime }$ to $V_{j}$ are the size of the quantum key of ground station $g_{i}$ stored in satellite $s_{j}$.

$$C_{V_{i}^{\prime },V_{j}}\;=\;Key^{Pool}\left(s_{j},g_{i}\right)$$

The capacities of the edges from $V_{S}^{\prime }$ to $V_{1}^{\prime }$ and $V_{2}^{\prime }$, $C_{V_{S}^{\prime },V_{1}^{\prime }},C_{V_{S}^{\prime },V_{2}^{\prime }}$, are the maximum forward quantum keys of ground stations $g_{1}$ and $g_{2}$.

$$C_{V_{S}^{\prime },V_{i}^{\prime }}\;=\;\sum_{V_{k}\in V}C_{V_{i}^{\prime },V_{k}},i\;=\;1,2$$

The capacity of the edge from $V_{T}^{\prime }$ to $V_{3}^{\prime }$ is the maximum forward quantum keys of ground station $g_{3}$.

$$C_{V_{T}^{\prime },V_{3}^{\prime }}=\sum_{V_{k}\in V}C_{V_{3}^{\prime },V_{k}}$$

The capacities of the edges from $S^{\prime }$ to $V_{S}^{\prime }$ and $V_{T}^{\prime }$ are the maximum sizes of the required quantum keys.

$$C_{S^{\prime },V_{T}^{\prime }}=C_{S^{\prime },V_{S}^{\prime }}\;=\;\min\left(R_{1}\;+\;R_{2},R_{3}\right)$$

where $R_{j}\left(j\;=\;1,2,3\right)$ is the required size of the quantum key of ground station $g_{j}$, and it is set manually. Lastly, the capacity of the edge from $V_{8}$ to $T^{\prime }$ is the sum of $C_{S,V_{T}^{\prime }},C_{S,V_{S}^{\prime }}$.

$$C_{V_{8},T^{\prime }}\;=\;C_{S^{\prime },V_{T}^{\prime }}+C_{S^{\prime },V_{S}^{\prime }}$$

**Figure 7:**
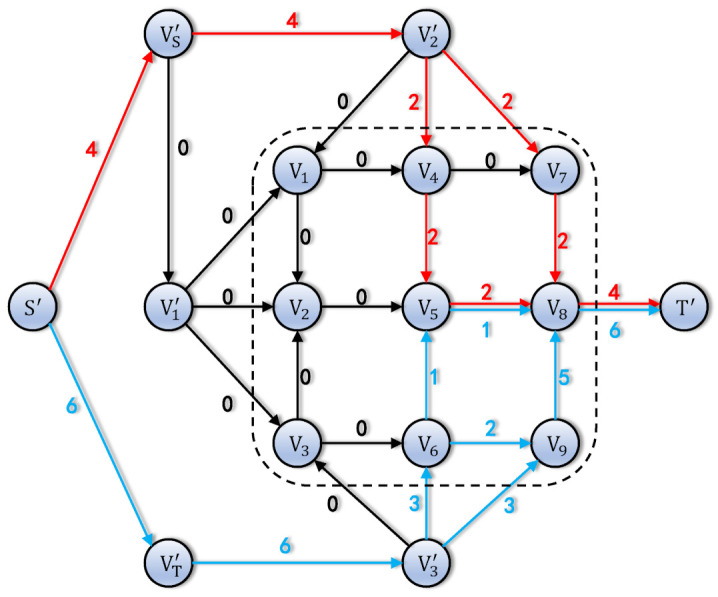
The max flow of auxiliary graph. The lines with same color represent the quantum key flow of same ground stations. The occupied traffic is drawn near the edges.

The Ford–Fulkerson method is the classical method to calculate the max flow in graph theory. By assigning the source and sink nodes, the max flow of $G^{\prime }$ is calculated as shown in Figure 7.

In the network traffic, there are no quantum keys of $g_{1}$ to be forwarded, due to the exhausted inter-satellite links. The traffic from $S^{\prime }$ to $V_{S}^{\prime }$ and $S^{\prime }$ to $V_{T}^{\prime }$ is not equal. So, the forwarded quantum keys of $g_{3}$ are decreased to be equal to the sum of the quantum keys of $g_{1}$ and $g_{2}$. It can be solved by modifying the required key sizes.

Algorithm 2 is proposed to calculate the forwarding paths and corresponding flows.
**Algorithm 2** Forwarding path algorithm.**Input:** Satellite $s^{*}$ and ground station $g^{*}$, key sizes of each inter-satellite link, required key sizes $R_{g},1\;\leq \;g\;\leq \;N_{gnd}$ and maximum hops *H*.**Output:** Flow distribution from other satellites to $s^{*}$.  Generate graph *G* according to the key sizes of inter-satellite links.  **for**
i←1
$N_{gnd}$
**do**   Add auxiliary node $V_{i}^{\prime }$   **for** j←1
**to**
$N_{sat}$ **do**     **if** $Hop(s^{*},j)\;\leq \;H$ **then**          Add edge that starts at node $V_{i}^{\prime }$ and ends at node $V_{j}$. The corresponding capacity is $Key^{Pool}\left(j,i\right)$.    **end if**  **end for****end for**Add auxiliary node $V_{S}^{\prime }$, edges $\left(V_{S}^{\prime },V_{i}^{\prime }\right),i\;=\;1,2,\cdots ,N_{gnd},i\;\neq \;g^{*}$ and capacities $C_{V_{S}^{\prime },V_{i}^{\prime }}\leftarrow \sum_{Hop(s^{*},j)\;\leq \;H}Key^{Pool}\left(j,i\right)$Add auxiliary node $V_{T}^{\prime }$, edges $\left(V_{T}^{\prime },V_{g^{*}}^{\prime }\right)$ and capacity $C_{V_{T}^{\prime },V_{g^{*}}^{\prime }}\leftarrow \sum_{Hop(s^{*},j)\;\leq \;H}Key^{Pool}\left(j,g^{*}\right)$.Add auxiliary node $S^{\prime }$, edges $\left(S^{\prime },V_{S}^{\prime }\right)$ and $\left(S^{\prime },V_{T}^{\prime }\right)$ and capacities $C_{S^{\prime },V_{S}^{\prime }},C_{S^{\prime },V_{T}^{\prime }}\leftarrow K\;=\;\min\left(\sum_{i\;\neq \;g^{*}}R_{i},R_{g^{*}}\right)$.Add auxiliary node $T^{\prime }$, edge $\left(V_{s^{*}},T^{\prime }\right)$ and capacity $C_{V_{s^{*}},T}\leftarrow 2K$.Calculate the flow distribution with maximum flow by Ford-Fulkerson method.Let $C_{S^{\prime },V_{S}^{\prime }},C_{S^{\prime },V_{T}^{\prime }}$ be the minimum flow of the edges $\left(S^{\prime },V_{S}^{\prime }\right)$ and $\left(S^{\prime },V_{T}^{\prime }\right)$Recalculate the flow distribution with maximum flow by Ford-Fulkerson method.

## 5. Simulation Results

In this section, simulations of accesses selection and routing are provided.

### 5.1. Scenario of Simulations

**Figure 8:**
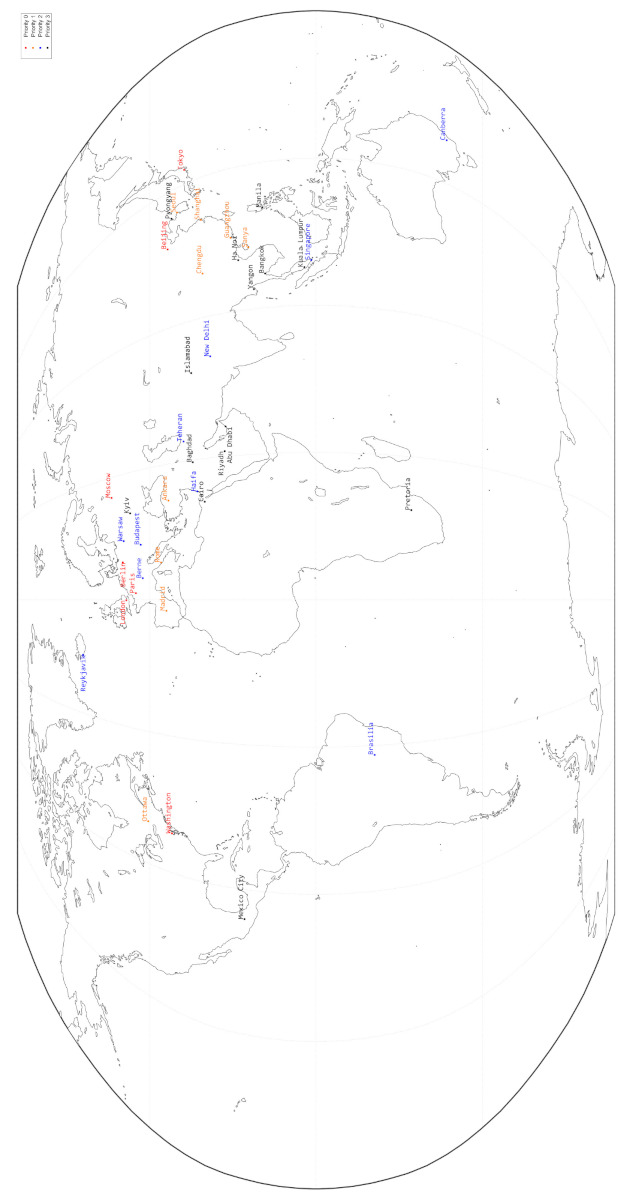
The distribution of ground stations.

The simulations consist of a constellation with 9 orbital planes and 40 ground stations located in 40 different cities globally. The distribution of ground stations are depicted in Figure 8.

The priorities (0 to 3, 0 for the highest priority, 3 for the lowest priority) of ground stations denote the weights of key sizes. Assume that the priorities of ground stations $g_{1}$ and $g_{2}$ are $p_{1}$ and $p_{2}$, respectively. The weight of the key sizes between $g_{1}$ and $g_{2}$ is $\max\{p_{1},p_{2}\}$. That is to say that the weight depends on the lower priority of the ground stations.

In each orbital plane, there are 40 satellites distributed evenly on each plane. The right ascension of the ascending nodes (RAAN) of these planes are distributed evenly from $45^{\circ }$ to $160^{\circ }$ within the interval step of $15^{\circ }$. $S_{j,k}$ is the *k*-th (k = 1,2,⋯, 40) satellite located in the *j*-th (j = 1,2,⋯, 9) plane. The classical parameters of $S_{j,k}$ are

(1) $$\begin{array}{ccc} a_{j,k} & \;=\; & R_{e}\;+\;h \end{array}$$

(2) $$\begin{array}{ccc} e_{j,k} & = & 0 \end{array}$$

(3) $$\begin{array}{ccc} i_{j,k} & \;=\; & 97.4902^{\circ } \end{array}$$

(4) $$\begin{array}{ccc} \Omega_{j,k} & \;=\; & 45.6^{\circ }+\left(j\;-\;1\right)\;\times \;15^{\circ } \end{array}$$

(5) $$\begin{array}{ccc} \omega_{j,k} & \;=\; & 0 \end{array}$$

(6) $$\begin{array}{ccc} \theta_{j,k} & \;=\; & k\frac{360^{\circ }}{40}\;+\;\left(j\;-\;1\right)\;\times \;1.4^{\circ } \end{array}$$

where h = 523.3 km is the altitude of orbits. The orbital epoch is 1 January 2022 00:00:00 UTCG. The orbital planes are all circular sun-synchronous orbits (SSO) with a revisit period of 7 days.

Furthermore, owing to the assumption that QKD is not implemented under daylight, the satellite-to-ground QKD is inactive for the satellites whose RAANs are $0^{\circ }$, $30^{\circ }$ and $175^{\circ }$. Hence, the constellation does not cover the whole Earth’s surface.

### 5.2. Key Sizes with Various Meteorological Conditions

The most significant factor to lower the key sizes of satellite-to-ground QKD is meteorological conditions. However, it is difficult to simulate meteorological system due to the chaotic nature. In the simulations, randomly generated meteorological loss is adopted to simulate the meteorological system. For simplicity, meteorological loss varies from 0 to 1, which represents the key size ratio of the current meteorological condition to clear nights. The key rates on clear nights are calculated according to the finite-size effect [24].

### 5.3. Simulation Parameters

The protocol parameters, optimized according to [25,26], of satellite-to-ground and inter-satellite QKD are listed in Table 4.

The optical parameters are listed in Table 5. For the satellite-to-ground QKD, the optical parameters are the same as those of the Micius satellite [6]. For the inter-satellite QKD, the diameters of the telescopes are enlarged to increase the secure key rate.

Actually, there are few secure keys of the inter-satellite QKD generated in the simulation if the diameter of the telescope on the satellite is 300 mm. In particular, the inter-planar links are blocked during equinoxes due to scarce secure key generation rates. Therefore, the diameter of the telescope on the satellite (both the transmitter and receiver) is set to be 500 mm for the inter-satellite QKD. For the satellite-to-ground QKD, same with Micius experiment, the diameter of telescope on satellite is 300 mm. Although there is high cost for manufacturing, there is another consideration to use telescopes with different sizes. For example, assume that the size of a satellite is 1000 mm ×1000 mm ×1000 mm. Four telescopes with a 500 mm diameter can be installed on the surface, or nine telescopes with 300 mm diameter (ignoring margins). The performance of the satellite-to-ground QKD, inter-planar QKD and intra-planar QKD can be promoted and balanced by combining telescopes with different sizes. This can be investigated in future research.

### 5.4. Key Sizes of Inter-Satellite QKD

The inter-satellite QKD is divided into the intra-planar QKD and inter-planar QKD. The intra-planar QKD is implemented by the adjacent satellites in the same plane. The key sizes of the intra-planar QKD are almost constant, due to the stable relative positions. However, the key sizes of the inter-planar QKD vary according to the relative positions and solar position.

### 5.5. Accesses Selection Algorithm

The potential key sizes $PK_{g}$ and implemented key sizes $IK_{g}$ of ground station *g* are the sum of key sizes from the set of the potential and selected accesses between satellites and ground station *g*, respectively.

$$PK_{g}\;=\;\sum_{a:Gnd(a)\;=\;g,a\;\in A}Key\left(a\right)$$

$$IK_{g}\;=\;\sum_{a:Gnd\left(a\right)\;=\;g,a\;\in A^{*}}Key\left(a\right)$$

where *A* represents the set of potential accesses and $A^{*}$ is the set of selected accesses. *A* contains the accesses that satellites in the constellation pass for all ground stations, without taking constraints into consideration. $A^{*}$ consists of the selected accesses that are the solution of Algorithm 1. Hence, potential key size $PK_{g}$ indicates how many secure key sizes are generated between the constellation and ground station *g* if there are no constraints. Implemented key size $IK_{g}$ denotes how many secure key sizes are actually implemented according to the solution of Algorithm 1.

**Figure 9:**
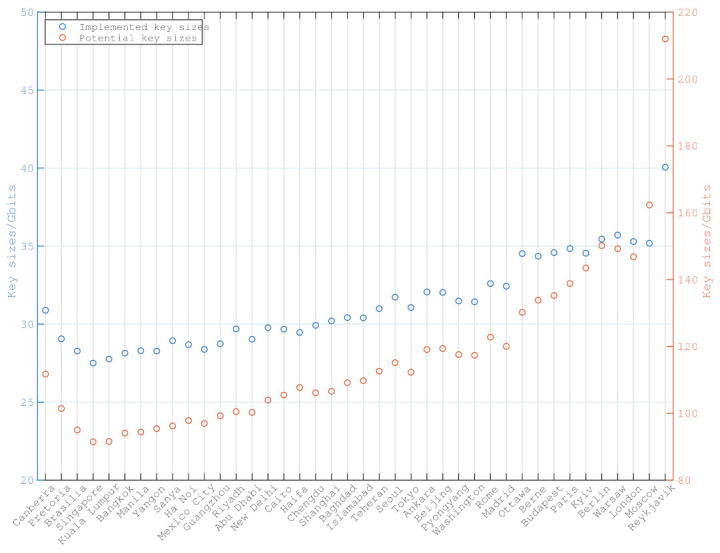
The implemented and potential key sizes between each ground station and satellites in constellation.

Figure 9 illustrates the potential and implemented key sizes of each ground station. Due to the pointing constraints and limited payloads, the implemented accesses are fewer than the potential accesses. Hence, the scale of the right y-axis is greater than the left.

**Figure 10:**
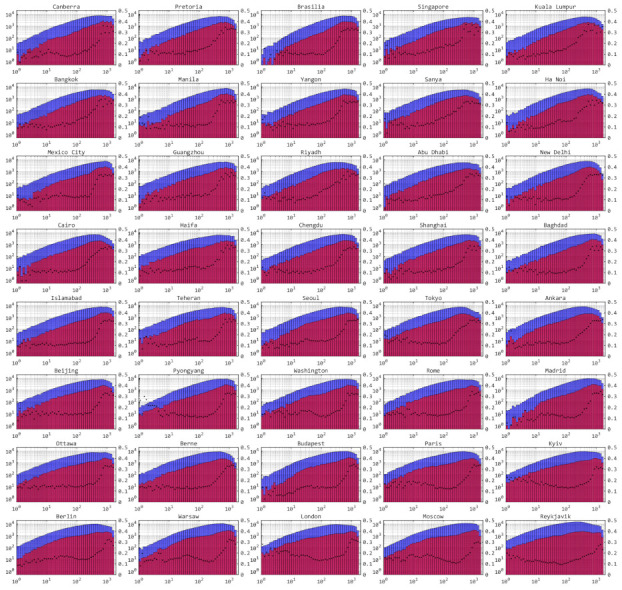
Histograms of potential and implemented key size distribution. The x-axes are key sizes of accesses. Left y-axes are numbers of corresponding accesses and right y-axes are ratios of implemented and potential numbers of accesses. The blue parts are the number of potential accesses, and red parts are the number of selected accesses. The black dots are the ratios.

Figure 10 describes the key size distributions of each access between the satellites and each ground station and the ratios of the implemented and potential numbers of accesses. Meteorological conditions are taken into consideration. Clearly, the numbers of accesses with higher key sizes are greater than those with lower key sizes. As the ratios of implemented and potential numbers of accesses increase with the key sizes, according to the accesses selection algorithm, the accesses with greater key sizes are more likely to be selected.

### 5.6. Routing Algorithm

**Figure 11:**
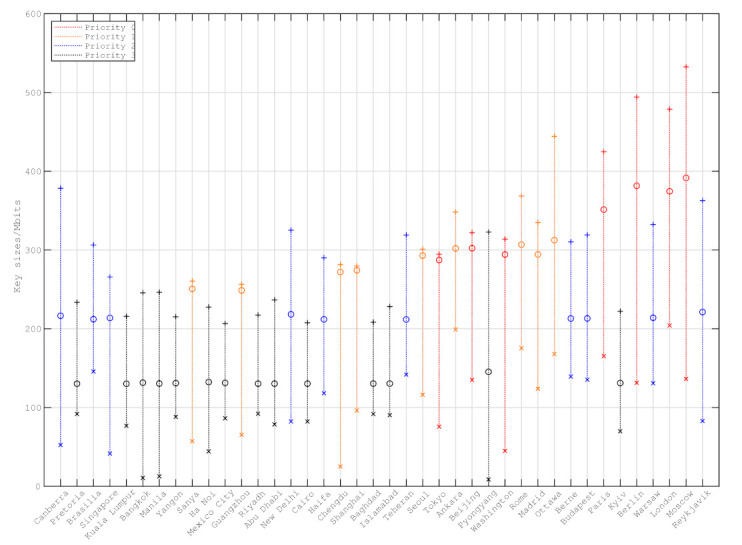
The average, maximum and minimum daily key sizes distributed by satellites of constellation to each ground station during a year.

Figure 11 shows the daily key sizes distributed by satellites of the constellation to each ground station. There are little fluctuations between the average daily key sizes of ground stations with priority 2 and 3. It is because there are enough quantum keys generated between the constellation and ground stations to be distributed. However, due to requiring more keys, there are significant differences between the average daily key sizes of ground stations with priority 0 and 1. The average daily key sizes of some ground stations with priority 0, e.g., Tokyo, Beijing and Washington, are even lower than the those of ground stations with priority 1. For such ground stations, it is helpful to improve their performance by adding QKD receivers.

**Figure 12:**
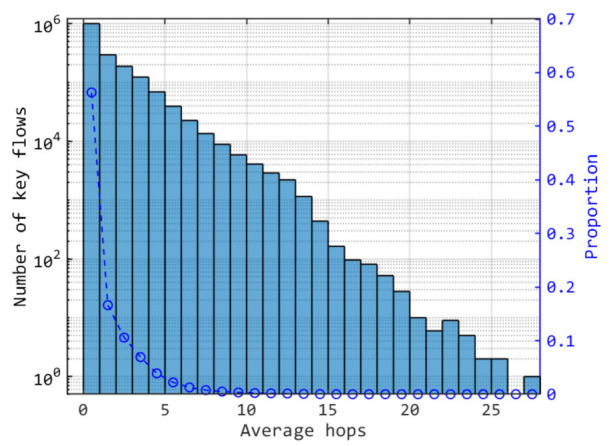
Distribution of key flows number with the average hops. The proportions are number of the corresponding key flows to total.

Figure 12 indicates the number distribution of key flows with the average hops. Average hops are ratios of key sizes consumed by inter-satellite links and key sizes distributed to ground stations. For the key flows with no consumption, the average hop is 0. In Figure 12, the proportion of key flows with no consumption is about 56%. That is to say that most distributed keys are relayed within one hop.

**Figure 13:**
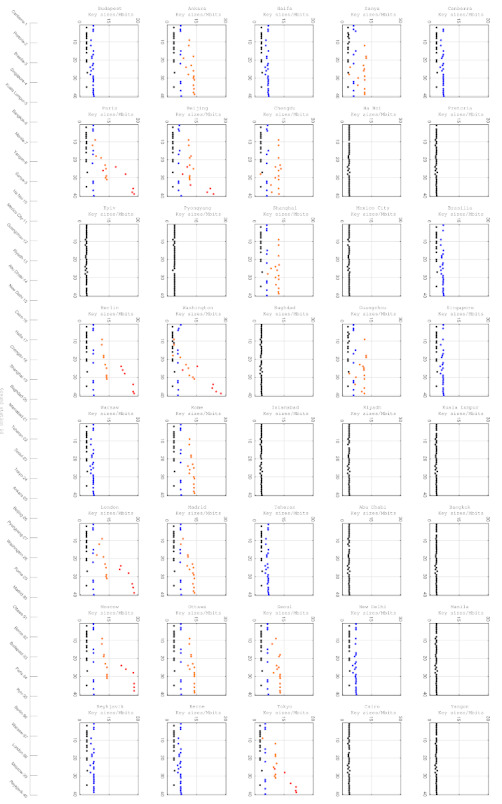
Average daily key sizes distributed by constellation for each ground station. Red dots represent the ground stations with priority 0, orange dots with priority 1, blue dots with priority 2 and black dots with priority 3. The ids of ground stations are sorted by ascending order of the latitudes of the ground stations.

Figure 13 is the key sizes distributed for each ground stations. For the ground stations with priorities 2 or 3, the average daily key sizes distributed vary smoothly. However, due to the high requirements of key sizes for ground stations with priorities 0 or 1, the performance is not good for the ground stations in a low latitude area.

**Figure 14:**
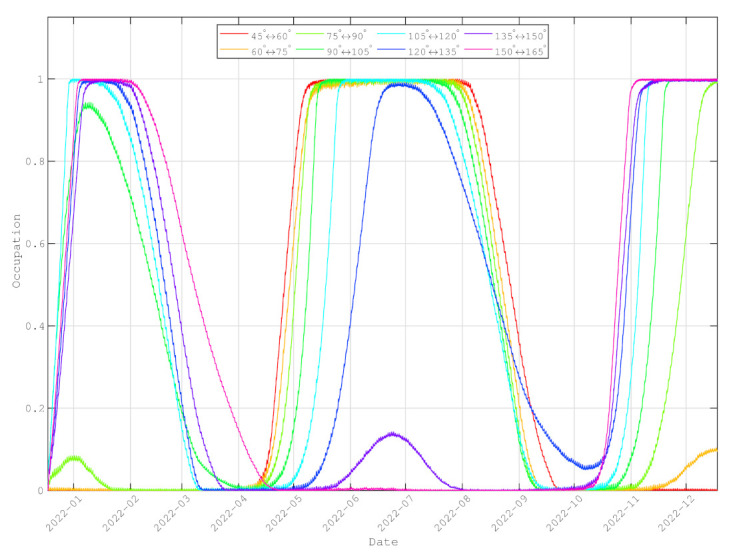
The average key size occupancy of each inter-planar link.

Figure 14 shows the average occupancies of inter-planar links during one year. For single inter-satellite links between satellite $s_{i},s_{j}$, the occupancy $Occ_{s_{i},s_{j}}$ is

$$Occ_{s_{1},s_{2}}\;=\;\frac{Key_{s_{1},s_{2}}^{ISL}}{Key_{max}^{ISL}}$$

where $Key_{max}^{ISL}$ is the maximum storage capacity of satellite per link. In the simulation, $Key_{max}^{ISL}\;=\;2Gb$ for each link and the initial storage is 0. The average occupancies of inter-planar links are the mean values of all the occupancies of the links that connect the two planes. The average occupancies of the intra-planar links are the mean values of all the occupancies belonging to the same plane.

The initial state of each satellite’s key pool is empty. However, the occupancies increase to their peaks, as the quantum key rate generated in January is high. A similar condition occurs when in June. For most inter-plana links, the generated key sizes increase to their peaks when near solstices. However, they decrease to their bottoms when near equinoxes. Such periodicity is related to solar motion. Note that the QKD under the Sun is close in our simulation. Therefore, the generation of quantum keys is dramatically affected by the solar influence. However, as the channels losses under the Sun are greater than the limitations, the key generation rates may be negative. In other words, no secure keys can be generated when the QKD is in daylight. Consequently, the inter-planar links become the networking bottlenecks periodically.

**Figure 15:**
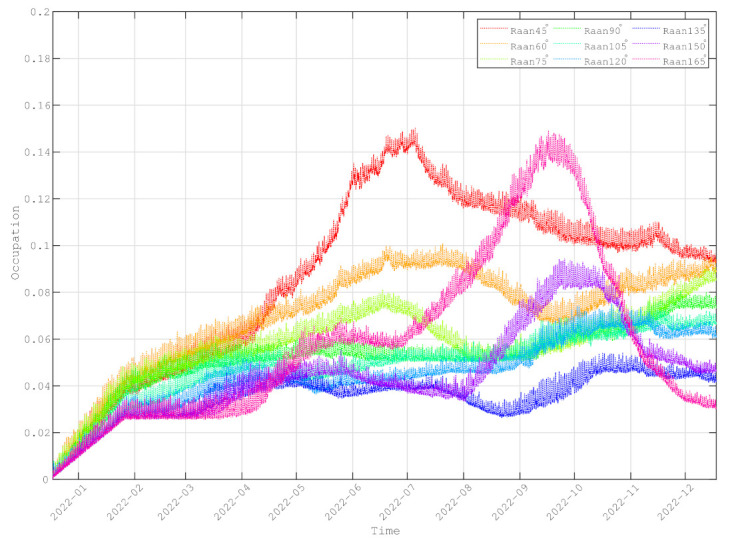
The average key size occupancy of each intra-planar link.

Figure 15 shows the average occupancies of the intra-planar links during one year. The average occupancies of the intra-planar links are lower than 20%. For some intra-planar links, their occupancies may approach 0%. Unlike the inter-planar links, the key generation rates of the intra-planar links are free from the Sun’s influence. Hence, the key generation rates are always stable. However, owing to the long channels distances, the key generation rates are not great enough to support the requirements of routing. Therefore, the intra-planar links are always the networking bottlenecks.

## 6. Discussion

In this paper, two important issues, accesses selection and inter-satellite routing, in the operation of the QKD constellation are investigated.

The first issue, accesses selection, decides whether or not an access is implemented according to the status of satellites and ground stations. The aim is to achieve greater key sizes generated between satellites and ground stations. The second issue, inter-satellite routing, finds a forwarding path for each key flow by taking constraints into consideration.

In accesses selection, the method for solving the longest path problem in DAG is applied so that the set of accesses in the longest path is chosen to be implemented. The simulation results show that the accesses selection algorithm proposed in this paper is helpful for choosing accesses with higher key sizes.

In inter-satellite routing, by finding a maximum flow in DAG, the maximum flow is the forwarding path. The simulation results show that the bottleneck of the constellation performance is the generated key sizes of the inter-satellite links. Both the inter-planar links and intra-planar links are restricted by the Sun. The key generation rates of the inter-planar links are influenced by the Sun. Although the key generation rates of the intra-planar links are not affected by the Sun, the keys stored are not sufficient to relay quantum keys. Thus, the intra-planar links are always the networking bottlenecks, and the inter-planar links are the networking bottlenecks periodically.

The straight method to promote the performance of inter-satellite links is decreasing the link distances. On one hand, in each orbital plane, there are only 40 satellites orbiting at an altitude of 523.3 km. The distances between adjacent satellites are about 1083 km. Therefore, it is effective to promote the networking performance of intra-planar links by adding satellites in the same plane. For example, the distances decrease to 600 km if there are 72 satellites. On the other hand, to improve the generated key rate of the inter-planar links, it is better to decrease the differences of RAANs of adjacent orbital planes. However, the coverage areas of the constellation are narrowed. In that case, more satellites are required to achieve networking globally.

Note that the above method of decreasing the link distances increases the relay nodes. Therefore, the consumed keys to be delivered from one ground station to another remote one are also increased. The trade-off needs to be studied in the future. Additionally, the following are the limitations of the study:

- The simulation of meteorological conditions is rough, as the meteorological conditions are static in each access, and the corresponding data are generated randomly.
- Only the single-layer LEO constellation is taken into consideration. The research of multiple-layer constellation is to be studied in detail.
- The networking traffic balance is not taken into consideration. Hence, some inter-satellite links may become bottlenecks if they are unavailable.
- The key flows are always routed from satellites with higher hops to lower. Although the consumed keys increase, the performance of the constellation may be improved if the key flows can be routed from satellites with lower hops to higher ones.
- Channel security and communication attacks are not taken into consideration in this study.

To sum up, the Sun is the key factor that restricts the networking of the quantum constellation. In this study, the coverage of the constellation is restricted due to the Sun. Furthermore, the key generation rates of inter-planar links are affected significantly by the Sun. Consequently, the keys that are delivered to a remote ground station decrease when inter-planar links have low efficiency. Although the key generation rates of intra-planar links are almost not influenced by the Sun, the rates are low due to the long link distances. Adding more satellites also probably increases the consumption of the relayed keys.

## Figures and Tables

**Table 1:** The definitions of symbols used in accesses selection problem.

Symbols	Definitions
$N_{sat}$	$N_{sat}\in Z^{+}$, number of satellites.
$N_{gnd}$	$N_{gnd}\in Z^{+}$, number of ground stations.
*A*	$A=\left\{a=\left(i,sat,gnd,st,et,key,ips,ipg,fps,fpg\right)\right\}$, set of accesses.
*i*	i∈{1,2,⋯,|A|}, index of access.
sat	$sat\in \{1,2,\cdots ,N_{sat}\}$, satellite of access.
gnd	$gnd\in \{1,2,\cdots ,N_{gnd}\}$, ground station of access.
st	$st\in R^{+}$, starting time of access.
et	$et\in R^{+}$, et>st, ending time of access.
key	$key\in R^{+}$, key size of access.
ips	$ips\in R^{4}$, pointing state of satellite at starting time.
ipg	$ipg\in R^{4}$, pointing state of ground station at starting time.
fps	$fps\in R^{4}$, pointing state of satellite at ending time.
fpg	$fpg\in R^{4}$, pointing state of ground station at ending time.
Index(a)	Index:A→{1,2,⋯,|A|}, denotes the index of access *a*.
Sat(a)	$Sat:A\to \{1,2,\cdots N_{S}\}$, denotes the satellite of access *a*.
Gnd(a)	$Gnd:A\to \{1,2,\cdots N_{G}\}$, denotes the ground station of access *a*.
St(a)	$St:A\to R^{+}$, denotes the starting time of access *a*.
Et(a)	$Et:A\to R^{+}$, denotes the ending time of access *a*.
Key(a)	$Key:A\to Z^{+}$, denotes the key size of access *a*.
Ips(a)	$Ips:A\to R^{4}$, denotes the pointing states of satellite of access *a* at starting time.
Ipg(a)	$Ipg:A\to R^{4}$, denotes the pointing states of ground station of access *a* at starting time.
Fps(a)	$Fps:A\to R^{4}$, denotes the pointing states of satellite of access *a* at ending time.
Fpg(a)	$Fpg:A\to R^{4}$, denotes the pointing states of ground station of access *a* at ending time.
$T(p_{1},p_{2})$	$T:R^{4}\times R^{4}\to R^{+}$, denotes the minimum transition time from pointing states $p_{1}$ to $p_{2}$.
$F(a_{i},a_{j})$	F:A×A→{0,1}, denotes whether transits from the accesses $a_{i}$ to $a_{j}$
	1 denotes true, 0 otherwise.

**Table 2:** The definitions of symbols used in inter-satellite routing.

Symbols	Definitions
$N_{sat}$	Number of satellites.
$N_{gnd}$	Number of ground stations.
*S*	Set of satellites, $S=\{s_{1},\cdots ,s_{N_{sat}}\}$.
*G*	Set of ground stations, $G=\{g_{1},\cdots ,g_{N_{gnd}}\}$.
$Hop(s_{i},s_{j})$	$Hop:S\times S\to Z^{+}$, denotes the minimum hop count from satellite $s_{i}$ to $s_{j}$.
$Key^{ISL}\left(s_{i},s_{j}\right)$	$Key^{ISL}:S\times S\to Z^{+}$, denotes the key sizes generated between satellites $s_{i}$, $s_{j}$.
$Key^{Pool}\left(s,g\right)$	$Key^{Pool}:S\times G\to Z^{+}$, denotes the key sizes generated
	between satellite *s* and ground station *g*.

**Table 3:** The network status.

Satellite		Key Pool	
$S_{1}$	$G_{1}$:0	$G_{2}$:5	$G_{3}$:2
$S_{2}$	$G_{1}$:0	$G_{2}$:3	$G_{3}$:0
$S_{3}$	$G_{1}$:4	$G_{2}$:4	$G_{3}$:0
$S_{4}$	$G_{1}$:0	$G_{2}$:0	$G_{3}$:4
$S_{6}$	$G_{1}$:3	$G_{2}$:0	$G_{3}$:0
$S_{7}$	$G_{1}$:0	$G_{2}$:0	$G_{3}$:6
$S_{9}$	$G_{1}$:3	$G_{2}$:0	$G_{3}$:0

**Table 4:** The protocol parameter of satellite-to-ground QKD.

Qkd Type	State	Parameter	Value
Satellite-to-ground QKD	Signal	Mean photons	0.46
		Probability	0.74
	Decoy	Mean photons	0.07
		Probability	0.20
	Vacuum	Probability	0.06
Inter-satellite QKD	Signal	Mean photons	0.48
		Probability	0.85
	Decoy	Mean photons	0.05
		Probability	0.11
	Vacuum	Probability	0.04

**Table 5:** The system parameters.

System	Parameter	Value	Unit
Satellite-to-ground	Diameter of telescope on ground (receiver)	1.0	[m]
	Diameter of telescope on satellite (transmitter)	300	[mm]
	Detector efficiency	0.5	[-]
	Optical efficiency	0.16	[-]
Inter-satellite	Diameter of telescope of receiver	500	[mm]
	Diameter of telescope of transmitter	500	[mm]
	Detector efficiency	0.5	[-]
	Optical efficiency	0.16	[-]

## References

[1] Bennett C.H., Brassard G., Breidbart S. (2014). Quantum Cryptography II: How to re-use a one-time pad safely even if P=NP. Nat. Comput..

[2] Elliott C. (2002). Building the quantum network. New J. Phys..

[3] Elliott C., Colvin A., Pearson D., Pikalo O., Schlafer J., Yeh H. (2005). Current status of the DARPA quantum network. Def. Secur..

[4] Dianati M., Alleaume R. Architecture of the Secoqc Quantum Key Distribution network. Proceedings of the 2007 First International Conference on Quantum, Nano, and Micro Technologies (ICQNM’07).

[5] Alléaume R., Bouda J., Branciard C., Debuisschert T., Dianati M., Gisin N., Godfrey M., Grangier P., Länger T., Leverrier A. (2007). SECOQC White Paper on Quantum Key Distribution and Cryptography. arXiv.

[6] Liao S.K., Cai W.Q., Liu W.Y., Zhang L., Li Y., Ren J.G., Yin J., Shen Q., Cao Y., Li Z.P. (2017). Satellite-to-ground quantum key distribution. Nature.

[7] Liao S.K., Cai W.Q., Handsteiner J., Liu B., Yin J., Zhang L., Rauch D., Fink M., Ren J.G., Liu W.Y. (2018). Satellite-Relayed Intercontinental Quantum Network. Phys. Rev. Lett..

[8] Sabuncu M., Ladislav Mišta J., Fiurášek J., Filip R., Leuchs G., Andersen U.L. (2007). Nonunity gain minimal-disturbance measurement. Phys. Rev. A.

[9] Sabuncu M., Filip R., Leuchs G., Andersen U.L. (2010). Environmental Assisted Quantum Information Correction for Continuous Variables. Phys. Rev. A.

[10] Vergoossen T., Loarte S., Bedington R., Kuiper H., Ling A. (2020). Modelling of satellite constellations for trusted node QKD networks. Acta Astronaut..

[11] Polnik M., Mazzarella L., Carlo M.D., Oi D.K., Riccardi A., Arulselvan A. (2020). Scheduling of space to ground quantum key distribution. EPJ Quantum Technol..

[12] Wang J., Chen H., Zhu Z. (2021). Modeling research of satellite-to-ground quantum key distribution constellations. Acta Astronaut..

[13] Tanizawa Y., Takahashi R., Dixon A. A routing method designed for a Quantum Key Distribution network. Proceedings of the 2016 Eighth International Conference on Ubiquitous and Future Networks (ICUFN).

[14] Yang C., Zhang H., Su J. (2018). Quantum key distribution network: Optimal secret-key-aware routing method for trust relaying. China Commun..

[15] Liu X., Wang J., Li R., Zhang C. (2018). Security Analysis of Stochastic Routing Scheme in Grid-Shaped Partially-Trusted Relay Quantum Key Distribution Network. Chin. J. Electron..

[16] Wang Y., Zhao Y., Chen W., Dong K., Yu X., Zhang J. Routing and Key Resource Allocation in SDN-based Quantum Satellite Networks. Proceedings of the 2020 International Wireless Communications and Mobile Computing (IWCMC).

[17] Dequal D., Vidarte L.T., Rodriguez V.R., Vallone G., Villoresi P., Leverrier A., Diamanti E. (2021). Feasibility of satellite-to-ground continuous-variable quantum key distribution. npj Quantum Inf..

[18] Derkach I., Usenko V.C. (2021). Applicability of Squeezed- and Coherent-State Continuous-Variable Quantum Key Distribution over Satellite Links. Entropy.

[19] Ma X., Qi B., Zhao Y., Lo H.K. (2005). Practical decoy state for quantum key distribution. Phys. Rev. A.

[20] Ma X., Fung C.H.F., Razavi M. (2012). Statistical fluctuation analysis for measurement-device-independent quantum key distribution. Phys. Rev. A.

[21] Zhang S.J., Xiao C., Zhou C., Wang X., Yao J.S., Zhang H.L., Bao W.S. (2020). Performance analysis of continuous-variable measurementdevice-independent quantum key distribution under diverse weather conditions. Chin. Phys. B.

[22] Liao S.K., Yong H.L., Liu C., Shentu G.L., Li D.D., Lin J., Dai H., Zhao S.Q., Li B., Guan J.Y. (2017). Long-distance free-space quantum key distribution in daylight towards inter-satellite communication. Nat. Photonics.

[23] Wertz J.R. (1978). Spacecraft Attitude Determination and Control.

[24] Zhang Z., Zhao Q., Razavi M., Ma X. (2017). Improved key-rate bounds for practical decoy-state quantum-key-distribution systems. Phys. Rev. A.

[25] Xu F., Xu H., Lo H.K. (2014). Protocol choice and parameter optimization in decoy-state measurementdevice-independent quantum key distribution. Phys. Rev. A.

[26] Zhu J.R., Li J., Zhang C.M., Wang Q. (2017). Parameter optimization in biased decoy-state quantum key distribution with both source errors and statistical fluctuations. Quantum Inf. Process..

